# The Peculiarities of the Acoustic Waves of Zero-Order Focusing in Lithium Niobate Plate

**DOI:** 10.3390/s21124000

**Published:** 2021-06-10

**Authors:** Iren Kuznetsova, Ilya Nedospasov, Andrey Smirnov, Vladimir Anisimkin, Dmitry Roshchupkin, Maria-Assunta Signore, Luca Francioso, Jun Kondoh, Mikhail Serebrov, Vadim Kashin, Vladimir Kolesov

**Affiliations:** 1 Kotelnikov Institute of Radio Engineering and Electronics of RAS, 125009 Moscow, Russia; ianedospasov@mail.ru (I.N.); andre-smirnov-v@yandex.ru (A.S.); anis@cplire.ru (V.A.); argentumv@yandex.ru (M.S.); vadim_kashin@mail.ru (V.K.); kvv@cplire.ru (V.K.); 2 Institute of Microelectronics Technology and High-Purity Materials Russian Academy of Sciences, 142432 Chernogolovka, Russia; rochtch@iptm.ru; 3 CNR, Institute for Microelectronics and Microsystems, 73100 Lecce, Italy; mariaassunta.signore@cnr.it (M.-A.S.); lucanunzio.francioso@cnr.it (L.F.); 4 Graduate School of Science and Technology, Shizuoka University, Shizuoka 432-8561, Japan; kondoh.jun@shizuoka.ac.jp

**Keywords:** focalized acoustic beams, plate acoustic waves of zero-order, lithium niobate plate, arc-like electrodes, interdigital transducer, slowness curves

## Abstract

In this research, beam focusing in lithium niobate plate was studied for fundamental anti-symmetric (A_0_) and symmetric (S_0_) Lamb waves, and the shear-horizontal (SH_0_) wave of zero-order. Using the finite element method, appropriate configuration of the interdigital transducer with arc-like electrodes was modeled accounting for the anisotropy of the slowness curves and dispersion of the modes in the plate. Profiles of the focalized acoustic beams generated by the proposed transducer were theoretically analyzed. Based on the result of the analysis, relevant delay lines were fabricated and transfer functions (insertion loss) of the line were measured for SH_0_ wave in YX-lithium niobate plate. Using an electron scanning microscope, distribution of the electric fields of the same wave were visualized. The results of this study may be useful for hybrid devices and sensors combining nano and acoustoelectronic principles.

## 1. Introduction

In recent years, hybrid devices combining nanoelectronic, acoustoelectronic, and microfluidic principles have been actively developed [1,2,3,4,5]. One issue arising from this research is the enforcement of acoustic wave power at a nanostructure location or inside a microfluidic channel. This problem may be solved by using focusing interdigital transducers (IDTs) to generate the acoustic wave.

At present, a large number of IDT configurations for the focusing of surface acoustic waves (SAWs) exist [6,7,8,9,10,11,12,13,14,15,16]. Most of these are based on the principle of bending electrodes, for which the geometrical focus depends on the shape, number, and period (the wavelength) of the electrodes. The operation of transducers has been studied both for piezoelectric [6,7,8,9,10] and piezosemiconductor materials [11,12]. SAW focusing has been demonstrated using wide-band phase array [6], arc-like IDTs [7,13,14], and ring-shaped electrodes [15,16]. Numerical analyses of the generated acoustic beams have used the Huigence principle [8], parabolic presentation of the slowness curve [9], two-dimensional surface Green-function method [10], three-dimensional finite element model (FEM) of a coupled field [13,14], and numerical models accounting for the curvature of the SAW slowness curves [17,18,19,20,21].

Focused SAWs are generated by IDTs with bent electrodes sending a wave to a point or narrow zone (focus). As a result, different modes are joined within some focal region, which is shifted from geometrically centered IDTs with constant radii. Due to crystal anisotropy, the propagation velocity and diffraction of the as-generated waves are significantly changed with the propagation direction. As a result, the generation of narrow acoustic beams using arc-like IDTs is realtively difficult. To overcome this problem, the curvature of the IDTs may be adapted to the relevant slowness curves [18,22,23,24] to provide time correlation of all waves propagating from different parts of electrodes to the focal point, and to ensure efficient wave interference in the geometrical center of the IDT. Transducers that focus SAWs operate from 40 to 500 MHz.

Detection and visualization of SAW beams generated by focusing IDTs has been undertaken using the laser Doppler vibrometer [17], thin liquid film deposition on waveguide [25], and scanning electron microscopy (SEM) [26,27].

The importance of the acoustic wave focusing method is also related to the microfluidic technique. For example, SAWs are actively used for remote micromanipulation in microhydrodynamic systems. SAW displacements generate a wave in microchannels filled with a liquid. Propagation of the wave in liquid leads to a liquid stream, which affects the particles in the liquid through resistance force. Acoustic radiation guides the particles along time-averaged pressure gradients that have the same scale as the particles [28,29,30,31]. As a result, the location of the focal point is critically important for the moving particles because the aperture of the acoustic beam is only minimal at the focal point. The focusing IDTs have demonstrated their reliability as microelements in microfluidics, in which they are applied to sleeping and producing drops, moving particles, etc. [32,33,34].

As indicated in the above review, effort in developing focusing methods has been largely concentrated on high-frequency waves, whereas papers devoted to focusing low-frequency waves, e.g., zero-order modes in piezoelectric plates, are practically absent. In some of these, special lenses such as phonon crystal elements were suggested to focus anti-symmetric zero-order Lamb waves [35]. In [36], theoretical analysis and experimental verification were undertaken of zero-order Lamb wave focusing by AlN membrane. Thus, it should be noted that zero-order Lamb waves focusing is not a trivial problem because the waves are characterized both by anisotropy and geometrical dispersion. Nevertheless, these waves may have an unusually high electromechanical coefficient and electromechanical transduction compared to SAWs in the same crystal [37,38].

In the present paper, the topology of the focusing interdigital transducer is theoretically analyzed, taking into account the anisotropy of the slowness curves for anti-symmetric (A_0_) and symmetric (S_0_) Lamb waves, in addition to the zero-order shear-horizontal (SH_0_) wave, in lithium niobate plate. Moreover, for the SH_0_ wave, an appropriate IDT was fabricated and tested. Experimental results were in good agreement with theoretical data.

First, the possibility of focusing fundamental zero-order waves in a lithium niobate plate using IDT electrodes with a constant radius of curvature was considered theoretically. Then, the slowness curves were calculated for these waves. To improve the focusing of the SH_0_ wave, IDT electrodes with a variable radius of curvature were proposed. Further, the proposed IDTs were manufactured and their characteristics measured.

## 2. Materials and Methods

### 2.1. Theoretical Methods

#### 2.1.1. FEM Simulation

For modeling acoustic wave focusing in a piezoelectric plate, two types of IDTs with bent electrodes were used. In one case, the fingers of the IDT had the form of circular arcs with the same radius, and in the other, the curvature of the bent electrodes varied in angle, taking into account the corresponding slowness curves.

**Figure 1:**
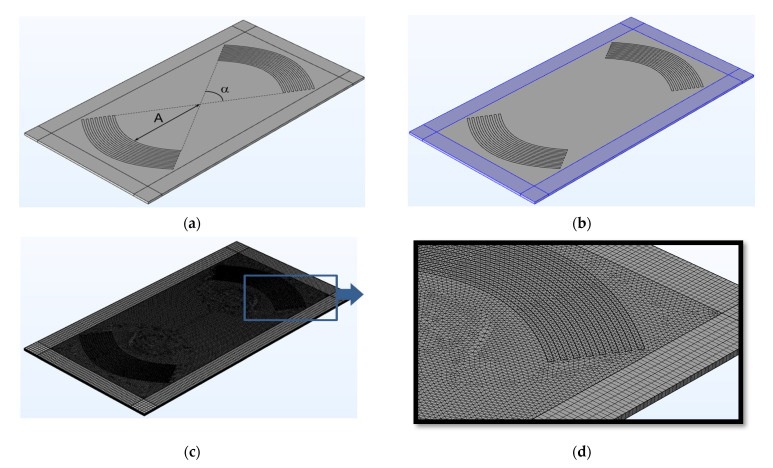
Theoretical model with three-angle grating in the bulk and rectangular grating in the ideally matched zone used for numerical modeling. (**a**) Topology of the FEM model, (**b**) PML location, (**c**) specified mesh of the model, and (**d**) enlarged fragment.

The geometry of the focusing phase grating is presented in Figure 1. In both cases, the IDTs contained 4 pairs of aluminum electrodes with thickness 400 nm placed on the surface of a lithium niobate plate with a thickness 0.305 mm. The width of each IDT finger and the distance between them was equal to *λ*/4, where *λ* is the wavelength. The distance (A) from the first IDT electrode to the geometric focus and the maximum arc opening angle (*α*) were equal to 10λ and 60°, respectively. The problem of exciting acoustic waves by these IDTs was solved numerically using the finite element method in the COMSOL Multiphysics environment. Two types of the meshes were used in the model: tetrahedral mesh for the region of the plate under the electrodes, and rectangular mesh for the area of the perfectly matching layers. The linear size of the mesh elements was chosen in the range from λ/5, as the maximum element size, to λ/10, as the minimum element size.

In the model, an acoustic wave was excited by applying an alternating electric voltage to the IDT electrodes. The mechanical load from the electrodes’ material was not taken into account due to its small thickness. The ideally matched layers placed on the edges of a plate were used to suppress wave reflections from the plate boundaries (Figure 1b). In the LiNbO3/vacuum interface, mechanical and electrical boundary conditions for the free surface were used. In the calculations, we used the material constants for lithium niobate given in [39].

#### 2.1.2. Boundary Transfer Matrix Method

**Figure 2:**
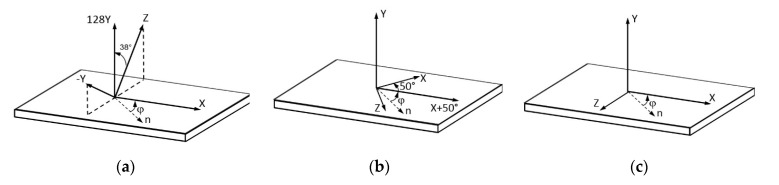
Crystallographic orientation of LiNbO_3_ plates analyzed in the paper (**a**) 128 YX, (**b**) Y − X + 50°, and (**c**) YX. *n* is the wave propagation direction.

The slowness curves 1/*V*(*φ*) of the waves under study were calculated using an iterative algorithm for the angle *φ* in conjunction with the conventional method of the transfer matrix [40], where *V* is the wave phase velocity. The crystallographic situations used in the calculations of these curves for A_0_, S_0_, and SH_0_ waves in a lithium niobate plate are shown in Figure 2 [41]. In the selected directions, these waves are characterized by the highest value of the electromechanical coupling coefficient [37].

### 2.2. Experimental Methods

#### 2.2.1. Production of Focusing IDT

An experimental sample of the acoustic delay line was fabricated using the common photolithography process and magnetron sputtering. The plate of YX LiNbO_3_ (East Freqcontrol Holding Co., Hangzhou, China), 305 μm thick, 18 mm wide, and 35 mm long—was used as a substrate with one polished face. By spinning, the polished surface was coated with positive photo-resistant S1813SP15 (Frast-M, Moscow, Russia), with a thickness of 2 μm. Then, the sample was heated (90 °C) for 30 min Hei-Tec (Heidolph Instruments GmbH&Co, Schwabach, Denmark) and the relevant mask was exposed on the surface of the sample covered with a photo-resistant coating SmartPrint setup (Microlight 3D, Lyon, France). The zone of the photo-resistance targeted by the IDTs was removed using special solvent P-236A-MF (Frast-M, Moscow, Russia), resulting in a photo-resistant mask to be targeted for electrode sputtering.

#### 2.2.2. Study of S-Parameters

The study of the frequency dependence of IDT transmission parameter S12 for the produced delay line was investigated in the range of 0.5–10 MHz. The S parameters were measured with a vector network analyzer (Tektronix TTR 506A, Beaverton, OR, USA). To calibrate the device, we used a calibration kit BN 533844 N CALKIT 50 MALE with 0 to 9 GHz (SPINNER, Munich, Germany). The vector analyzer was connected to the contact pads of the IDTs using indium and gold wires with diameter of 25 μm and length of 30 mm.

#### 2.2.3. Visualization of Acoustic Wave Propagation

To verify the focusing ability of the transducers, a scanning electron microscope was used and the electric field distribution of the acoustic wave generated by the transducer was visualized [26,27]. The detector of the microscope, which was registered with low-energy secondary electrons with energy of 1 eV, allowed visualization of the acoustic wave fields on the surface of piezoelectric crystals because the electrons were sensitive to the electric fields that accompanied the acoustic wave propagation.

## 3. Results and Discussion

### 3.1. Theoretical

#### 3.1.1. IDT Electrodes with a Constant Angle Radius of Curvature

First, the acoustic fields of A_0_, S_0_, and SH_0_ waves in a lithium niobate plate excited by focusing IDTs, in which the electrodes had a constant radius of curvature of angle *φ*, were calculated. In this case, the distance *A* from the first electrode to the center of the plate was equal to the curvature of the electrodes *R* = 10*λ* at *φ* = 0 [17]. *λ* was equal to 1.227, 1.412, and 1.057 mm for A_0_, S_0_, and SH_0_ waves, respectively.

**Figure 3:**
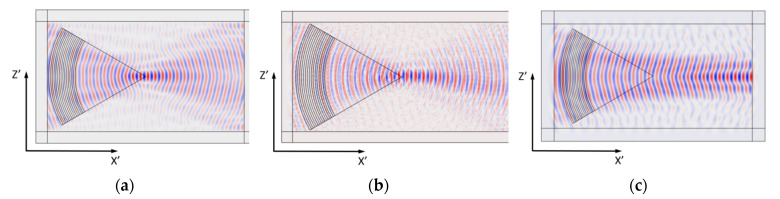
Distribution of the mechanical displacement component *U_Z’_* in the direction of wave propagation X’ for: (**a**) A_0_ (128YX, *f* = 1.849 MHz), (**b**) S_0_ (Y − X + 50°, *f* = 4.889 MHz), and (**c**) SH_0_ (YX, *f* = 3.7 MHz) waves in a lithium niobate plate (*h* = 0.305 mm), excited by electrodes with a constant radius with the angle *φ*.

Distributions of the mechanical displacement components *U_Z’_* in the direction of the wave propagation X’ for A_0_, S_0_, and SH_0_ waves propagating in 128YX, Y − X + 50°, and YX lithium niobate plates, and at the frequencies *f* = 1.849, 4.889, and 3.7 MHz, respectively, are presented in Figure 3. Note that the Z’ and X’ axes correspond to the rotated coordinate systems.

Comparison of the results obtained for the waves under study showed that in the case of Lamb waves A_0_ and S_0_, good focusing was achieved when using electrodes with a radius constant at the angle *φ*. In the case of the SH_0_ wave, poor focusing was observed.

**Figure 4:**
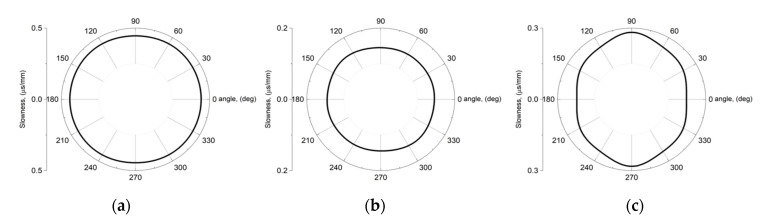
Slowness curves for acoustic plate waves in LiNbO_3_ crystal: (**a**) A_0_ wave at *hf* = 564 m/s on plane of 128Y-cut, (**b**) S_0_ wave at *hf* = 1488.9 m/s on plane of Y-cut, (**c**) SH_0_ wave at *hf* = 1300 m/s on plane of Y-cut. Zero angles 0° refer to Figure 2. *h* is the plate thickness.

It can be seen that the dependence of the phase velocity of the A_0_ wave on the angle *φ* is practically absent and the slowness surface can be considered (Figure 4a). In this case, the directions of energy transfer and wave propagation coincide. This explains the good focusing of the acoustic field of the A_0_ wave by IDT electrodes with a constant radius of curvature at angle *φ* (Figure 3a). Figure 3a also shows that the location of the focal point coincides with the center of the substrate.

For the S_0_ wave, slight anisotropy of the phase velocity was found. In Figure 4b, an angle of 0° corresponds to the direction of propagation X + 50°. Near this direction, the slowness curve can be considered to be close to isotropic (Figure 4b). Nevertheless, for IDTs with a large opening angle *α* > 60°, which are used to excite the S_0_ wave in a given crystallographic situation, poor focusing can be expected.

Analysis of Figure 4c shows the presence of strong anisotropy of the phase velocity when the wave vector rotates around the crystallographic axis Y. In this case, the direction along the center of the plate corresponds to the crystallographic axis X (Figure 2c). It can be seen that near the direction of wave propagation to the X axis, there is a slight negative concavity on the slowness curve (Figure 4c). It should be noted that such a negative curvature itself contributes to the focusing of the energy flux because the normal to the slowness curve corresponds to the direction of the wave energy flux. However, as shown by calculations, the use of IDT electrodes with a constant radius of curvature at the angle φ for focusing SH_0_ is ineffective. In this regard, it was decided to take into account the effect of the anisotropy of the slowness curve for the SH_0_ wave on the radius of curvature and the period of the focusing IDT.

#### 3.1.2. The Effect of Anisotropy of the Phase Velocity and Geometric Dispersion

As mentioned above, the anisotropy of the phase velocity of an acoustic wave in a plate at a constant frequency has a strong effect on the parameters of focusing electrodes [17]. In this work, to calculate the radius of curvature of the electrode *R* and the period of the IDT as a function of the angle φ for the SH_0_ wave, the following expression was used [17]:

(1) $$R\left(\varphi \right)=L_{0}\frac{V\left(\varphi \right)}{V\left(\varphi =0\right)}.$$

where *L*_0_ = *m × λ*(*φ*), *m* is number of wavelength, *λ*(*φ*) = *V*(*φ*)/*f*, *φ* is the angle from the direction of propagation X in the Y plane (Figure 2c). It should be noted that when expanding Expression (1) in a Taylor series in the approximation of smallness of the angle and taking into account terms up to the second order inclusive, it is possible to obtain:

(2) $$R\left(\varphi \right)=L_{0}\left(1+\frac{\gamma }{1+\gamma }\frac{\varphi^{2}}{2}\right).$$

where *γ* is the anisotropy coefficient.

A comparative analysis of the calculation results obtained using these formulas for evaluating the excitation of focused surface acoustic waves (SAWs) by curved transducers was carried out in [17]. It was shown that the most effective SAW focusing is obtained by approximating the curvature of the focusing IDTs using Formula (1). In addition, using (1) to estimate the distance between the electrodes allows the dependence of the wavelength on the angle of deviation from the direction of wave propagation along the center of the plate to be easily obtained:

(3) $$R\left(\varphi \right)=\frac{V\left(\varphi \right)}{f_{0}}.$$

where *f_0_* is the frequency of the wave, which is set by the IDT period and is constant. To calculate the radius of curvature of the IDT electrodes, depending on the angle *φ*, 120 points were selected on the slowness curve (Figure 4c) in the range from 30° to 330° clockwise, i.e., the maximum value of *α* was 60°. Then, using Expressions (1) and (3), and these points, the curvature of all electrodes and the period of the IDT were calculated. The distance A from the first electrode to the center of the plate, as in the case of electrodes with a constant radius, was equal to 10λ. It should be noted that due to anisotropy, the geometric focus of an IDT with a constant radius and the focus of an IDT with a varying radius will be located at different points, despite the fact that they have the same distance *A*.

**Figure 5:**
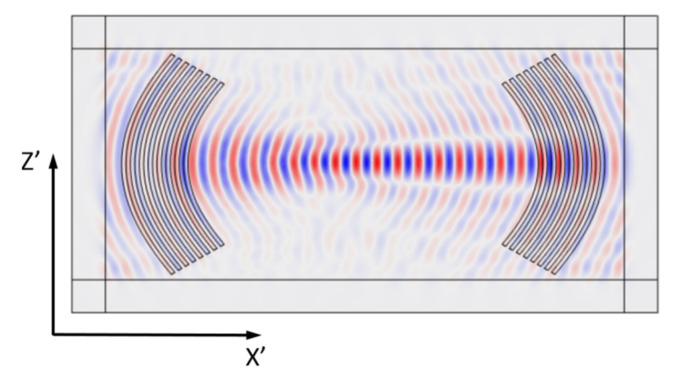
Distribution of the mechanical displacement component *U_Z’_* in the direction of wave propagation X’ for SH_0_ wave in YX LiNbO_3_ at *f* = 3.7 MHz excited by electrodes with a curvature that takes into account the anisotropy in the angle *φ*.

Figure 5 presents the results of the calculation of the distribution of the mechanical displacement component *U_Z’_* in the direction of propagation X’ of the SH_0_ wave excited by the IDT with an angularly varying radius in a lithium niobate plate with a thickness of *h* = 0.305 mm at a frequency of *f* = 3.7 MHz. In this case, *λ* was equal to 1.057 and 0.97 mm for *φ* = 0° and 30°, respectively. The focusing of the acoustic field and the plane wave front in the middle of the plate are clearly visible.

**Figure 6:**
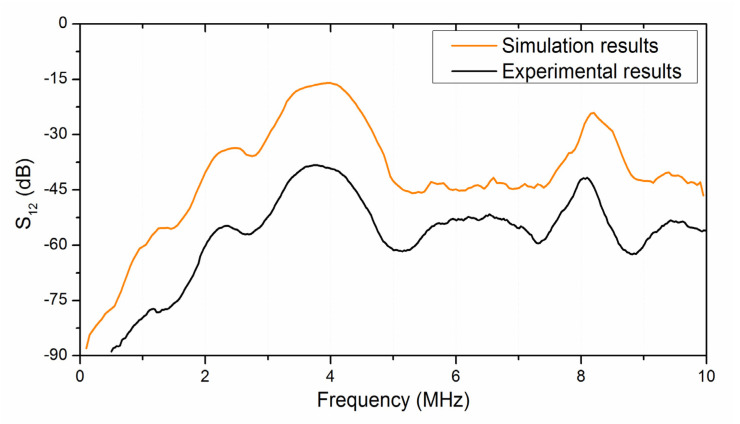
Frequency dependencies of the transmission parameter S_12_ (insertion loss) as calculated (**red line**) and measured (**black line**) for the delay line with the focusing IDTs based on the SH_0_ wave in the YX LiNbO_3_ plate.

To calculate the amplitude–frequency characteristics of the proposed focusing IDTs, an identical receiving IDT was placed on the opposite side of the plate. The calculated frequency dependence of the transmission parameter S_12_ for a given delay line (red curve) is shown in Figure 6.

### 3.2. Experimental

**Figure 7:**
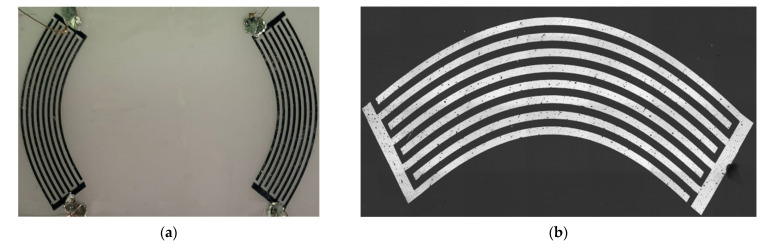
Photos of (**a**) the delay line with focusing IDT with electrodes with variable radius of curvature, and (**b**) enlarged IDT.

Based on the result of the modeling described above, a relevant photomask and delay line were fabricated. Photos of the focusing IDT and its view in a laser confocal Olympus microscope are shown in Figure 7a,b, respectively.

The frequency dependence of the produced delay line measured experimentally (black curve) is presented in Figure 6. Analysis showed that the SH_0_ wave is excited at a frequency of 3.752 MHz. Comparison of the theoretical and experimental dependences shows their good frequency agreement. We believe the disagreement in insertion loss is due to the mismatch in the impedance of IDTs with 50 ohms (resistivity of external circuits).

**Figure 8:**
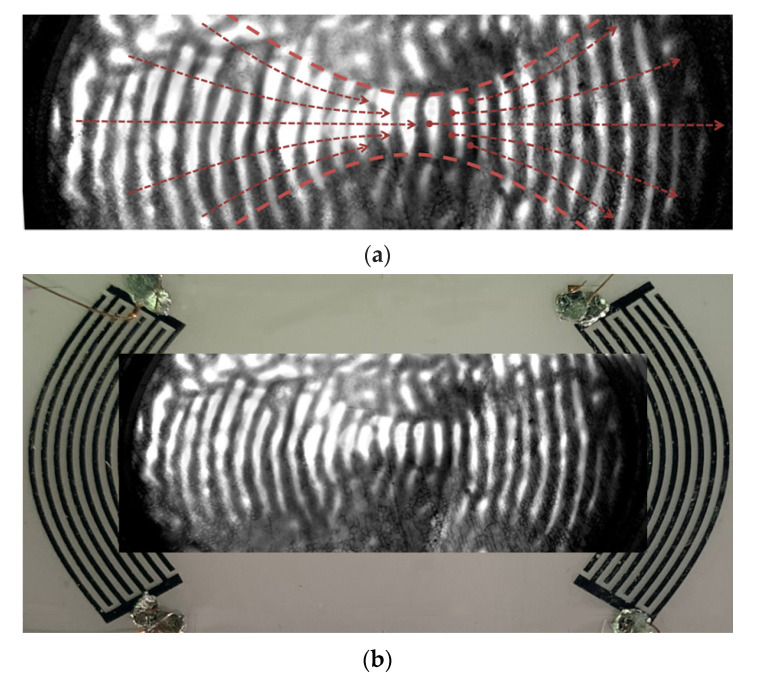
(**a**) Electric field of the SH_0_ wave on the surface of YX LiNbO_3_ plate visualized with an electron microscope and (**b**) optical photo of the delay line combined with the field image measured using the SEM method.

To experimentally confirm the focusing of the SH_0_ wave using a scanning electron microscope and a detector operating in the mode of recording low-energy secondary electrons, the distribution of the wave’s electric field between IDTs was recorded. Figure 8a shows a SEM image of the resulting electric field distribution. For clarity, Figure 8b shows an optical photograph that combines the delay line and the SEM image.

The resulting visualization confirms the conclusions of the theory and indicates the possibility of focusing SH_0_ waves with a very high electromechanical coupling coefficient in the center of the plate.

## 4. Conclusions

This work investigated the features of focusing antisymmetric (A_0_) and symmetric (S_0_) Lamb waves and waves with shear-horizontal polarization (SH_0_) of zero order in a piezoelectric lithium niobate plate. Using the finite element method, taking into account the anisotropy of slowness curves and mode dispersion in the plate, the design and shape of the corresponding focusing interdigital transducers (IDTs) were modeled. A theoretical analysis of the acoustic fields of the resulting focused wave beam was carried out. It was found that, in the case of strong wave velocity anisotropy, the IDT’s fingers with a variable radius of curvature can be used for effective acoustic field focusing. Based on theoretical calculations, a delay line was produced with curved electrodes for the excitation of SH_0_ waves in an YX LiNbO_3_ plate with thickness 0.305 mm, width 18 mm, and length 35 mm. The IDTs of the delay line contained four pairs of aluminum electrodes with thickness 400 nm. The width of each IDT finger and the distance between them was equal to *λ*/4, where *λ* is the wavelength. In the case of IDTs for exciting the focused SH_0_ wave, *λ* was equal to 1.057 and 0.97 mm for *φ* = 0° and 30°, respectively. The distance from the first IDT electrode to the geometric focus and the maximum arc opening angle were equal to 10*λ* and 60°, respectively. The work frequency of the produced delay line for the SH_0_ wave was 3.752 MHz. This was in a good agreement with theoretical calculations. Using a scanning microscope, the distribution of the SH_0_ wave electric field in the YX LiNbO_3_ plate produced by bent electrodes was studied. The possibility of SH_0_ wave effective focusing was confirmed. The results obtained will be useful in the development of hybrid nano-acoustoelectronic sensors and devices. We propose placing metal nanowires between the IDTs and increasing the value of the acoustoelectric effect due to focusing the electric field of the acoustic waves [1].
